# Following the Epidemic Waves: Child and Youth Mental Health Assessments in Ontario Through Multiple Pandemic Waves

**DOI:** 10.3389/fpsyt.2021.730915

**Published:** 2021-11-17

**Authors:** Shannon L. Stewart, Aadhiya S. Vasudeva, Jocelyn N. Van Dyke, Jeffrey W. Poss

**Affiliations:** ^1^Faculty of Education, University of Western Ontario, London, ON, Canada; ^2^School of Public Health Sciences, University of Waterloo, Waterloo, ON, Canada

**Keywords:** COVID-19, child and youth, mental health, interRAI, assessment, referrals, school closures

## Abstract

Emerging studies across the globe are reporting the impact of COVID-19 and its related virus containment measures, such as school closures and social distancing, on the mental health presentations and service utilization of children and youth during the early stages of lockdowns in their respective countries. However, there remains a need for studies which examine the impact of COVID-19 on children and youth's mental health needs and service utilization across multiple waves of the pandemic. The present study used data from 35,162 interRAI Child and Youth Mental Health (ChYMH) assessments across 53 participating mental health agencies in Ontario, Canada, to assess the mental health presentations and referral trends of children and youth across the first two waves of the COVID-19 pandemic in the province. Wave 1 consisted of data from March to June 2020, with Wave 2 consisting of data from September 2020 to January 2021. Data from each wave were compared to each other and to the equivalent period one year prior. While assessment volumes declined during both pandemic waves, during the second wave, child and youth assessments in low-income neighborhoods declined more than those within high-income neighborhoods. There were changes in family stressors noted in both waves. Notably, the proportion of children exposed to domestic violence and recent parental stressors increased in both waves of the pandemic, whereas there were decreases noted in the proportion of parents expressing feelings of distress, anger, or depression and reporting recent family involvement with child protection services. When comparing the two waves, while depressive symptoms and recent self-injurious attempts were more prevalent in the second wave of the pandemic when compared to the first, a decrease was noted in the prevalence of disruptive/aggressive behaviors and risk of injury to others from Wave 1 to Wave 2. These findings highlight the multifaceted impact of multiple pandemic waves on children and youth's mental health needs and underscore the need for future research into factors impacting children and youth's access to mental health agencies during this time.

## Introduction

Many children and families across the globe are now entering their second year of living in a pandemic (1) since the novel coronavirus (COVID-19) was first declared a pandemic by the World Health Organization (WHO) in March of 2020 (2). According to the WHO, even prior to the onset of this pandemic, an estimated 10–20% of children and youth were struggling with mental health problems globally (3), with self-harm being the third leading cause of death for adolescents in 2015 (4). Not only is the estimated prevalence of mental health disorders amongst children and youth on the higher end of this global estimate in Ontario, Canada, but an alarming number of these children and youth did not have their needs adequately met prior to the pandemic (5, 6). While an emerging body of cross-sectional studies globally are currently focusing on the mental health presentations of children and youth during periods of lockdown (7, 8), less attention has been afforded to referral and service utilization trends of service-seeking children and youth, which may constitute a particularly vulnerable group during this time.

School and non-essential service (e.g., childcare facilities, playgrounds, and recreational spaces) closures and limited in-person access to primary care and mental health settings during periods of government lockdowns, alongside widespread social distancing requirements, are expected to not only exacerbate mental health problems amongst a significant proportion of children and youth, but also potentially impact their access to timely mental health services (9–12). In Ontario, professionals in schools, childcare settings, and primary care settings are often involved in the identification of children and youth's mental health needs and subsequentially, providing the appropriate referrals to community mental health agencies (12). As a result of in-person closures of these settings, it is anticipated that many children and youth have been unable to access the appropriate mental health services during periods of government lockdown (12). However, there remains limited empirical data examining the anticipated changes in children's mental health assessments during the pandemic in Canada (13).

### Impact of School Closures on Child and Youth Mental Health

Considering that children and youth spend the majority of their time in school, or engaging in school-related activities, school closures have arguably had the greatest impact on their daily lives during this time (14). In addition to opportunities for academic growth, schools also provide children with needed structure and supports that, when disrupted, can impair sleeping patterns, reduce opportunities for physical activities, and limit the restriction of excessive caloric intake and leisure screen use (15), all of which are factors associated with children and youth's mental health and well-being (16–18). Disrupted routines and lack of structure themselves can also be extremely stressful for children and youth with neurodevelopmental disorders that are hypersensitive to changes (14, 19, 20) and for those with behavioral and emotional difficulties that rely on well-structured routines to help effectively cope with daily stressors and symptoms (14, 19).

Schools in North America also provide a continuum of mental health services to children (21). Schools can provide direct mental health services to children (6, 21) and some children rely solely on school-based mental health services to meet their needs, with a greater proportion of these children likely residing in low-income neighborhoods (22, 23). Moreover, some children may also be confined in unsafe homes where they are experiencing child abuse, maltreatment and/or neglect, including child exposure to domestic violence, without the support of a larger circle of adults (e.g., teachers, mental health professionals, and extended family and friends) that could potentially identify signs of abuse, intervene, and/or provide referrals to child welfare agencies (11, 14, 24).

On the other hand, for a subset of children, school closures can also have a positive impact on mental health outcomes. Considering that children and youth are now spending the majority of their time with family, their well-being during this time may be largely dependent on family functioning and their caregiver's abilities to help them effectively manage distressing feelings and emotions that might arise during the pandemic (11, 25). While for some children and youth, increased caregiver burden, distress, and conflict during this time might increase their own distress and inability to cope with pandemic-related stressors, others might benefit from the increased quality time with responsive caregivers that are able to adequately buffer some of their distress (11, 25). Additionally, school closures themselves might also result in temporary alleviation of school-related pressures such as social and performance anxiety (11, 26, 27), as well as a potential reduction stressors such as bullying (28).

### Impact of the Pandemic on Pediatric Mental Health Referrals and Related Admissions

Emerging studies globally are reporting a general decrease in pediatric mental health presentations to emergency departments and related hospital admissions and referrals to secondary mental health agencies amongst their samples during the early stages of the lockdown, when compared to either the months leading up to the pandemic or year prior as the pre-pandemic time frame (29–38). This is echoed in Ontario, Canada, where child and youth referrals to select mental health agencies dropped, on average, 38% during the first eight months of the pandemic when compared to the same months pre-pandemic (13). With Ontario currently experiencing its third wave, it remains unclear how the uncertainty and instability of the province moving in and out of lockdown is impacting service utilization and mental health presentations within this vulnerable population throughout the multiple waves. Moreover, although Ontario initially saw a reduction in emergency department visits and admissions related to self-harm for youth during the first wave (39), recent media stories highlight concerns for a rise in mental health related pediatric admissions and hospitalizations, specifically those related to self-harm, as the pandemic progresses into additional waves (40, 41). Therefore, more information is needed regarding the mental health presentations and service utilization of children and youth during multiple waves of the pandemic period.

### Current Study

The primary aim of this study was to examine how the volume of assessed children and youth within select mental health agencies in the Province of Ontario, Canada, changed during the distinct pandemic waves, which also correspond to a large degree with in-school learning closure. Using scales and algorithms drawn from standard care assessments within these agencies, we also sought to identify any significant changes in child and youth mental health presentations that occurred during the pandemic in order to identify some of the potential changes in the child and youth mental health system demands during an extraordinary period of societal crisis and uncertainty. A secondary aim of this investigation, extending upon the findings of our previous study (13), was to examine how more vulnerable communities, specifically families living within low-income neighborhoods, might be differentially impacted throughout the multiple waves (42, 43).

To address our research aims, we compared mental health presentations of treatment-seeking children and youth within the Province of Ontario across four timeframes: Wave 1, Wave 2, pre-pandemic comparison for Wave 1, and pre-pandemic comparison for Wave 2. We also utilized area-based measures of income to examine the associations between socioeconomic status and pandemic-related changes in the mental health presentations of children within more vulnerable communities that might be disproportionately impacted by pandemic-related stressors (42, 43).

Since schools play a critical direct and indirect role in the mental health needs and referrals of children, school closure timelines in Ontario were carefully considered in the determination of wave boundaries and the interpretation of our findings. While all students in Ontario were learning remotely from March 2020 to June 2020 (44), in September, all students had the option to resume in-person learning until January 2021 (45). During this time period, elementary schools re-opened full-time and secondary schools re-opened on a part-time capacity only (45). In January 2021, majority of students had to return to online learning due to rising cases of COVID-19 in the province, except in seven regions in Ontario (46).

While we anticipated an overall decline in referrals during the pandemic, we anticipated that this decline would be greater during periods of complete closure of in-person classes during the first wave, as compared to periods where students had the option of returning to school in-person during the second wave. Moreover, we anticipated that children and youth in low-income neighborhoods would experience the greatest decline in assessments overall.

## Materials and Methods

### Procedure

Data used for this study were drawn from assessments conducted as part of standard clinical practice at 53 mental health agencies in Ontario. These agencies assess individuals using the interRAI Child and Youth Mental Health (ChYMH) (47) full assessment, the interRAI Child and Youth Mental Health and Developmental Disabilities (ChYMH-DD) (48), or the interRAI ChYMH Screener (ChYMH-S) (49), described in greater detail below. As part of voluntary implementation of these assessments in the province, these agencies, representing ~70% of such agencies across Ontario, receive comprehensive training on how to administer and score these instruments and access to secure online software for recording of assessment responses. Although some clinicians might utilize the items on the ChYMH (45) as a part of their diagnostic decision-making, the interRAI ChYMH (45) is a needs-based, rather than diagnostic, assessment system that can be administered by a variety of mental health professionals working with children and youth (e.g., social workers, nurses, psychologists, or physicians) to support their data collection, care formulation, and subsequent care planning. Children and youth can be referred to these agencies through school settings, other professionals providing care to children and families (e.g., healthcare providers, children's aid societies, daycare settings, community centers), and self-referrals. These community-based agencies are funded by the provincial government and do not charge fees for their services.

Data used for this study comprised of all assessment records completed between March 1, 2019 and January 31, 2021 among agencies that conducted assessments throughout the time period examined in this study. These assessment events represent the flow of clinical cases through the agencies, and no additional exclusion criteria were applied in assembling the analytic dataset used in these analyses. Assessment licensing and data sharing agreements allow for the de-identified data to be uploaded onto interRAI's online data server that can be accessed by interRAI fellows for research purposes. InterRAI represents a collaborative network of researchers and practitioners. Both the first and last authors on this investigation are interRAI fellows (see interRAI.org). The Western University Research Ethics Board granted approval for all secondary analyses conducted in this investigation.

Further, we classified periods during the COVID-19 pandemic into two distinct waves: Wave 1 comprised the months of March through June 2020, and Wave 2 the months of September 2020 through January 2021. Boundaries of the waves are similar to other Ontario-based investigations (50, 51), with Wave 1 ending on June 30 and Wave 2 beginning on September 1, avoiding the months of July and August when COVID-19 cases were low (52) and the majority of students would not regularly attend school regardless of the pandemic. These boundaries also allowed for similarity of timeframes (i.e., comparing frames of four months and five months). Assessments completed during these two waves were compared with the equivalent periods 12 months earlier, and with each other, totaling to 35,162 assessments.

### Analytical Plan

Analytic measures were taken directly from these assessment instruments, along with computed scales and algorithms described below. Chi-square tests were used for significance testing between pandemic waves and the equivalent period one year earlier, as well as those comparing Wave 1 and Wave 2, with a significance level of 0.05. SAS, Version 9.4 for Windows was utilized to conduct all the statistical analyses for this paper. Copyright © 2013 SAS Institute Inc. SAS and all other SAS Institute Inc. product or service names are registered trademarks of SAS Institute Inc., Cary, NC, USA.

In addition, we used the first three digits of the child/youth's postal code (Forward Sortation Area, FSA) to link to public files of the 2016 Canadian census to inform neighborhood median household income for each FSA (53). Each FSA represents one of the 513 geographic areas in Ontario, designed for the administration of the postal system. We subsequently assigned these median incomes into quartiles. The national statistics agency compiles this information based on the total net income, after taxes, of related individuals residing in the same dwelling, and then calculates the median value within each FSA. Income Quartile 1 represents children living in neighborhoods with a median household income that falls within the lowest 25% of income in Ontario and income Quartile 4 represents children living in neighborhoods with a median household income that falls within the highest 25% incomes in Ontario (53).

All assessments were classified as being either a first assessment (of any kind) for the client by the agency, or a subsequent assessment. We chose to adjust for age, sex, neighborhood income, and whether a client was being assessed for the first time by an agency when comparing the pandemic period with the period one year prior. All assessments used in the analysis were assigned an adjusted weight, based on the inverse of the proportion in the pre-pandemic vs. pandemic periods, using the combination of these four measures. For example, if the proportion of males ages seven and under in Income Quartile 1 and new to the agency was 1.0% in the comparable pre-pandemic period, and 0.5% in the pandemic period, the pre-pandemic cases used a weight of one and the pandemic period cases used a weight of two to adjust for the decreased likelihood of selection of that type of case in the pandemic period.

Resulting *p*-values for all chi-square tests are presented without adjustment for multiple testing in the Supplementary Table 1. With many of the tested characteristics being correlated with each, other such adjustments may be too conservative. While standard 0.05 levels are used to denote significance it is recognized that there is a risk of results with *p*-values just below this threshold of having lower confidence.

### Assessment Tools

#### InterRAI Child and Youth Mental Health Assessment

The interRAI ChYMH (47) is a 400-item tool used in child and youth populations to obtain a data-driven picture of the individual's various mental health needs. Trained assessors complete this comprehensive assessment by consulting with multiple sources of information including the child or youth, caregivers, teachers, clinicians, and available medical and education records. The interRAI ChYMH is part of an integrated health information assessment system in which psychometrically sound scales and algorithms are embedded within the instrument to support clinicians in identifying the child's strengths and areas of risk and inform care planning (54–65).

#### InterRAI Child and Youth Mental Health and Developmental Disabilities Assessment

Similar to the ChYMH, the interRAI ChYMH-DD (48) provides a comprehensive, standardized, and empirically-supported mental health measure to support comprehensive care planning (66, 67), outcome measurement (62, 68); quality indicators (69) and case-mix classification (70) to estimate relative resource intensity (48, 65, 70). However, this measure is specifically intended for use with children and youth with developmental disabilities (48).

#### InterRAI Child and Youth Mental Health Screener

The interRAI ChYMH-S (49) is a short, 99-item assessment which complements the ChYMH full assessment and takes ~15 min to administer. The instrument aids assessors by assisting in decisions related to triaging, placement, and service utilization (71). It provides a brief snapshot of the child's level of functioning and assists clinicians to ascertain acuity levels, as well as in determining whether a more comprehensive assessment is needed (49, 72, 73).

### Scales and Algorithms

#### Risk of Suicide and Self-Harm in Kids (RiSsK)

The RiSsK algorithm is an empirically-supported decision making tool assessing the child's attempt to kill self, self-harm without attempt to kill, consideration of self-injury, others concerned about self-injury, family feeling overwhelmed, and any self-injurious behaviors (61). With a range of 0 to 6 and higher values denoting higher risk (61), this scale was dichotomized as 0 vs. 1 to 6.

#### Risk of Injury to Others (RIO)

The RIO is an empirically-support decision making tool which measures risk of harm to others in clinically-referred children and youth populations. The algorithm assesses violent ideation, threatened violence, violence to others, verbal abuse, socially inappropriate or disruptive behavior, family overwhelmed, impulsivity, and physical abuse^1^. With a range of 0 to 6 and higher values denoting higher risk^1^, this scale was dichotomized as 0 vs. 1 to 6.

#### Disruptive/Aggression Behavior Scale (DABS)

The frequency and severity of aggressive and disruptive behavior is assessed using the DABS. Items include physical abuse, verbal abuse, socially inappropriate or disruptive behavior, destructive behavior toward property, and outbursts of anger (54). With a range of 0 to 20 and higher values denoting greater severity of behaviors (54), this scale was dichotomized as 0 to 3 vs. 4 to 20.

#### The Depression Severity Index (DSI)

The DSI measures depressive symptoms in child populations including sad or pained facial expressions, making negative statements, self-deprecation, guilt/shame, and hopelessness (57, 74). With a range of 0 to 15 and higher values denoting greater depressive symptoms (57), this scale was dichotomized as 0 to 3 vs. 4 to 15.

#### Anxiety Scale

The anxiety Scale assesses anxiety systems through six items such as: anxious complaints or concerns, unrealistic fears, obsessive thoughts, intrusive thoughts or flashbacks, episodes of panic, and nightmares (62). With a range of 0 to 28 and higher values denoting higher levels of anxiety (62), this scale was dichotomized as 0 to 2 vs. 3 to 28.

#### Hyperactive/Distraction Scale (HDS)

Hyperactivity and distractibility are assessed by the empirically supported HDS. Items include impulsivity, ease of distraction, hyperactivity, and disorganization (54). With a range of 0 to 16 and higher values denoting higher risk (54), this scale was dichotomized as 0 to 8 vs. 9 to 16.

## Results

**Figure 1:**
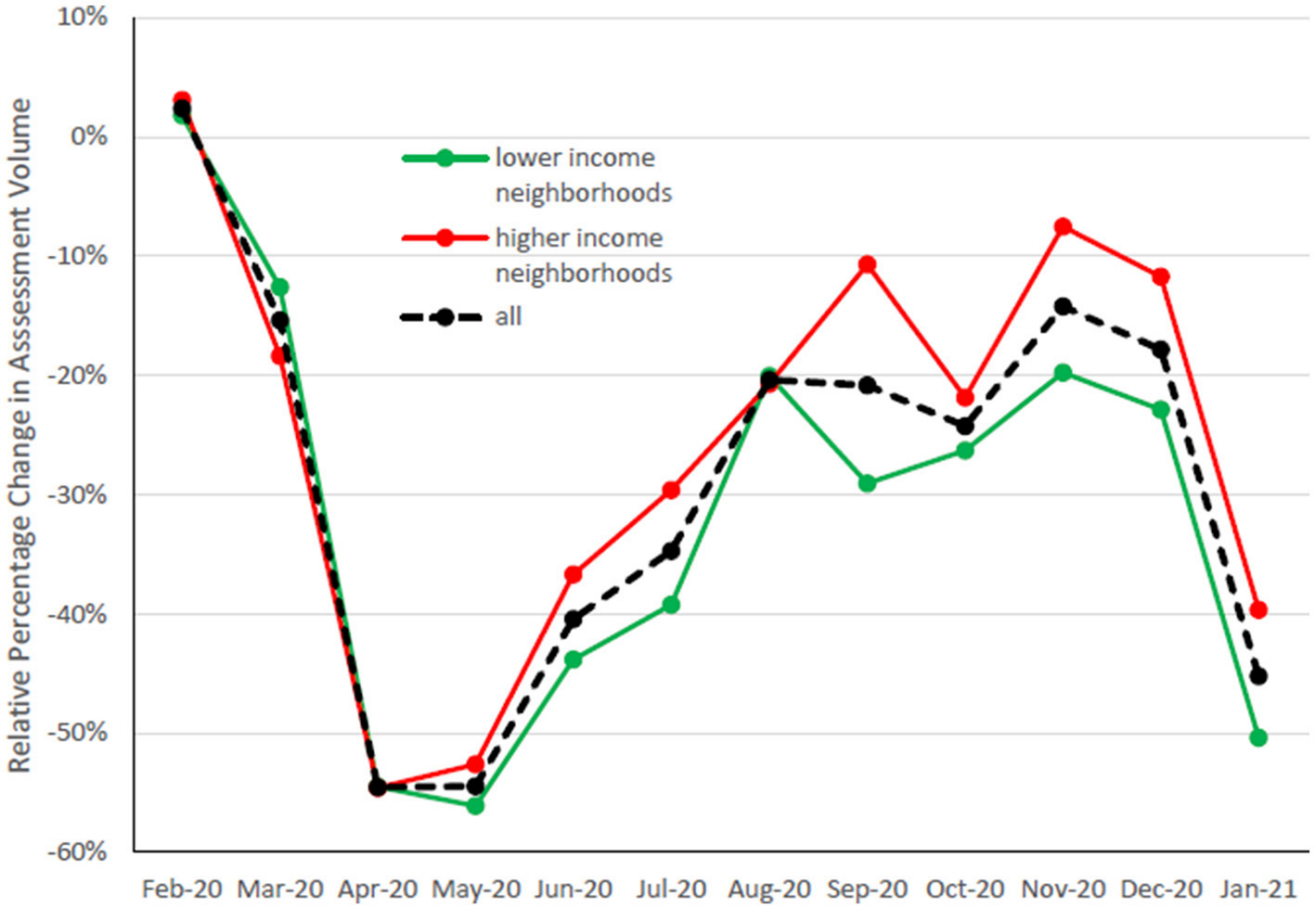
This represents the relative percentage change in assessment volumes for each month, as compared to the same month one year prior.

Shown in Figure 1, year-over-year assessment volume dropped by over 50% during the first two months of the pandemic, then recovered somewhat to about 20% lower by the start of the second wave in September, when in-class learning was occurring for some students. In January 2021, it plunged again to 45% below January 2020, a month when most of the province did not have in-class learning and when COVID-19 cases were recording new daily highs. Figure 1 also shows assessment volume change stratified by neighborhood income, split into those above and below the median; no significant difference in volume among income quartiles is seen in Wave 1 (*p* = 0.40), but in Wave 2, there was a greater reduction in assessments of children and youth from the two lower income quartile neighborhoods than those from the higher two income neighborhoods (*p* <0.0001). This Wave 2 difference can also be seen at the income quartile level in Table 1.

**Table 1:** Change in assessment volume, by characteristic, during wave 1 and wave 2, compared to one year prior.

	**Wave 1 (4 months)**				**Wave 2 (5 months)**			
**Characteristics^a^**	**Mar 2019**	**Mar 2020**	**% change**	***P* value**	**Sep 2019**	**Sep 2020**	**% change**	***P* value**
	**to Jun 2019**	**to Jun 2020**			**to Jan 2020**	**to Jan 2021**		
	**n (%)**	**n (%)**			**n (%)**	**n (%)**		
Total number of assessments (ChYMH, ChYMH-DD, and ChYMH-S)	9,953	5,690	−42.8%		11,176	8,343	−25.3%	
Age (years)								
7 & under	1,803 (18.1%)	944 (16.6%)	−47.6%	0.262	1,833 (16.4%)	1,067 (12.8%)	−41.8%^*^	<0.0001
8–11	3,392 (34.1%)	1,672 (29.5%)	−50.7%^*^	<0.0001	3,451 (30.9%)	2,327 (27.9%)	−32.6%^*^	<0.0001
12–15	3,567 (35.9%)	2,198 (38.7%)	−38.4%^*^	0.048	3,984 (35.7%)	3,376 (40.5%)	−15.3%^*^	<0.0001
16–21	1,181 (11.9%)	863 (15.2%)	−26.9%^*^	<0.0001	1,889 (16.9%)	1,560 (18.7%)	−17.4%^*^	0.001
Sex								
Males	5,253 (52.9%)	2,801 (49.3%)	−46.7%^*^	<0.0001	5,938 (53.2%)	3,780 (45.4%)	−36.3%^*^	<0.0001
Females	4,678 (47.1%)	2,876 (50.7%)	−38.5%^*^		5,221 (46.8%)	4,539 (54.6%)	−13.1%^*^	
First assessment for this client in this agency	6,470 (65.0%)	3,374 (59.3%)	−47.9%^*^		7,305 (65.4%)	5,132 (61.5%)	−29.7%^*^	<0.0001
Method of Assessment^b^								
Conducted in person	2,443 (83.6%)	422 (19.8%)	−82.7%^*^	<0.0001	2,791 (81.3%)	538 (16.7%)	−80.7%^*^	<0.0001
Phone	478 (16.4%)	1,255 (58.8%)	+162.6%^*^	<0.0001	629 (18.3%)	1,572 (48.8%)	+149.9%^*^	<0.0001
Video	2 (0.1%)	459 (21.5%)	+22,850%^*^	<0.0001	11 (0.3%)	1,114 (34.6%)	+10,027%^*^	<0.0001
Primary language								
English	9,388 (94.3%)	5,484 (96.4%)	−41.6%^*^	<0.0001	10,774 (96.4%)	8,019 (96.1%)	−25.6%	0.295
French	194 (1.9%)	84 (1.5%)	−56.7%^*^	0.031	175 (1.6%)	131 (1.6%)	−25.1%	0.981
Other	370 (3.7%)	121 (2.1%)	−67.3%^*^	< .0001	227 (2.0%)	192 (2.3%)	−15.4%	0.198
Area median household income quartile after tax								
1st < $57,367	2,910 (30.0%)	1,624 (28.8%)	−44.2%	0.356	3,406 (30.8%)	2,252 (27.3%)	−33.9%^*^	<0.0001
2nd $57,367 to $70,334	2,204 (22.7%)	1,307 (23.2%)	−40.7%	0.234	2,567 (23.2%)	1,912 (23.1%)	−25.5%	0.933
3rd $70,335 to $84,750	2,759 (28.4%)	1,613 (28.6%)	−41.5%	0.400	3,052 (27.6%)	2,407 (29.1%)	−21.1%^*^	0.018
4th > $84,750	1,828 (18.8%)	1,092 (19.4%)	−40.3%	0.203	2,048 (18.5%)	1,689 (20.4%)	−17.5%^*^	0.001

a *Some characteristics do not total 100% due to small number of missing responses*.

b *ChYMH and ChYMH-DD assessments only*.

* *Denotes significance, p <0.05*.

Table 1 presents the proportional change in year-over-year assessment volume in Wave 1 and Wave 2. In both waves, assessments of younger clients declined more than older ones and assessments of boys more than girls. Language (English/French/other) showed differing rates of change in Wave 1, but not in Wave 2. The proportion of assessments completed for clients new to an agency declined in both waves. Unsurprisingly, many more assessments were conducted by phone or by video, and fewer in-person, in both waves.

Table 2 presents the rate of a number of dichotomous measures for Wave 1 and Wave 2 and for the equivalent period one year earlier, adjusted for age, sex, neighborhood income and initial agency encounter. More prevalent in both waves were having witnessed domestic violence in the last year and having a parent experience a major life stressor in the last 90 days. Less prevalent in both waves were caregiver distress and having received child protection services in the last 90 days. Many more characteristics showed lower prevalence during Wave 2, compared to the year prior, including risk of harm to others, disruptive/aggressive behavior, hyperactivity, emotional abuse, youth justice involvement, drug use, child/youth having one parent as the legal guardian, family overwhelmed, and economic trade-offs. Higher prevalence in Wave 2 compared to the year prior were found for depressive symptoms and self-injurious attempts. Comparing the two waves, risk of injury to others, disruptive/aggressive behavior, involvement with youth criminal justice, and family overwhelmed were less prevalent in Wave 2, and depressive symptoms and a recent self-injurious attempt were more prevalent in Wave 2.

**Table 2:** Outcome scales and selected measures during wave 1 and 2 of the COVID-19 pandemic compared to the period one year prior – adjusted^**^.

**Outcome Scales and Selected Measures**	**Wave 1^a^**			**Wave 2^b^**			**Difference between Wave 1 and Wave 2**	
	**Period 1-year prior**	**COVID-19 period**	***P***	**Period 1-year prior**	**COVID-19 period**	***p***	***p***	**Change from Wave 1 to Wave 2**
Risk of Suicide and Self Harm in Kids (RiSsK) 1+	48.6%	49.6%	0.174	49.2%	49.7%	0.392	0.758	
Risk for Injury to Others (RIO) 1+	47.9%	47.0%	0.228	**48.1%**	43.3%	<0.0001^*^	<0.0001^*^	Decrease
Disruptive/Aggressive Scale (DABS) 4+	39.3%	38.8%	0.511	**39.2%**	36.1%	<0.0001^*^	0.002^*^	Decrease
Depressive Symptom Inventory (DSI) 4+	47.4%	48.7%	0.068	49.0%	**50.4%**	0.027^*^	0.015^*^	Increase
Anxiety Scale 3+	56.3%	57.4%	0.139	57.0%	57.9%	0.145	0.322	
Hyperactivity/Distractibility Scale (HDS) 9+	30.2%	31.4%	0.090	**30.8%**	29.6%	0.038^*^	0.100	
Witnessed domestic violence within last month	1.0%	1.2%	0.381	1.2%	1.1%	0.259	0.942	
Witnessed domestic violence within last year	5.0%	**5.9%**	0.007^*^	5.4%	**6.0%**	0.029^*^	0.997	
Experienced sexual assault/abuse within last year	2.2%	1.9%	0.239	2.3%	2.1%	0.198	0.734	
Experienced physical assault/abuse within last year	4.3%	4.6%	0.346	4.6%	4.0%	0.016^*^	0.088	
Experienced emotional abuse within last year	9.2%	9.9%	0.071	**10.4%**	9.5%	0.028^*^	0.776	
Self-injurious attempt in the last month	8.3%	7.8%	0.194	7.9%	**9.0%**	0.001^*^	0.010^*^	Increase
Referral as a result of involvement with youth justice system	5.1%	6.0%	0.135	**5.6%**	3.7%	<0.0001^*^	0.000^*^	Decrease
Street drug use (illegal or legal) last 14 days	5.8%	5.4%	0.285	**6.7%**	5.4%	0.001^*^	0.818	
Legal guardian mother or father only	29.5%	29.4%	0.812	**29.1%**	27.3%	0.002^*^	0.060	
Current custody dispute	5.3%	4.3%	0.060	4.2%	4.6%	0.448	0.487	
Parent/primary caregiver expresses feelings of distress, anger, or depression	**31.1%**	27.5%	0.002^*^	**34.0%**	25.8%	<0.0001^*^	0.184	
Family members report feeling overwhelmed	35.6%	36.0%	0.622	**37.8%**	33.5%	<0.0001^*^	0.008^*^	Decrease
Parent experienced major life stressor last 90 days	26.8%	**31.8%**	<0.0001^*^	26.9%	**29.1%**	0.042^*^	0.081	
Parental addiction in the last month	3.9%	3.4%	0.063	3.7%	3.6%	0.712	0.715	
Limited funds resulted in child/youth or parent making economic trade-offs	3.4%	2.6%	0.086	**3.6%**	2.6%	0.009^*^	0.715	
Child protection services received last 90 days	**21.1%**	17.4%	0.001^*^	**19.3%**	16.9%	0.004^*^	0.594	

*Bolded numbers denote the period with the higher % to indicate the direction of the change from the COVID-19 period and the period prior, where the difference is statistically significant*.

a *Wave 1 represents March 2020 to June 2020 (4 months)*.

b *Wave 2 represents September 2020 to January 2021 (5 months)*.

* *p <0.05*.

** *Wave 1 and Wave 2 columns adjusted for age, sex, neighborhood income, first encounter with the service agency*.

**Figure 2:**
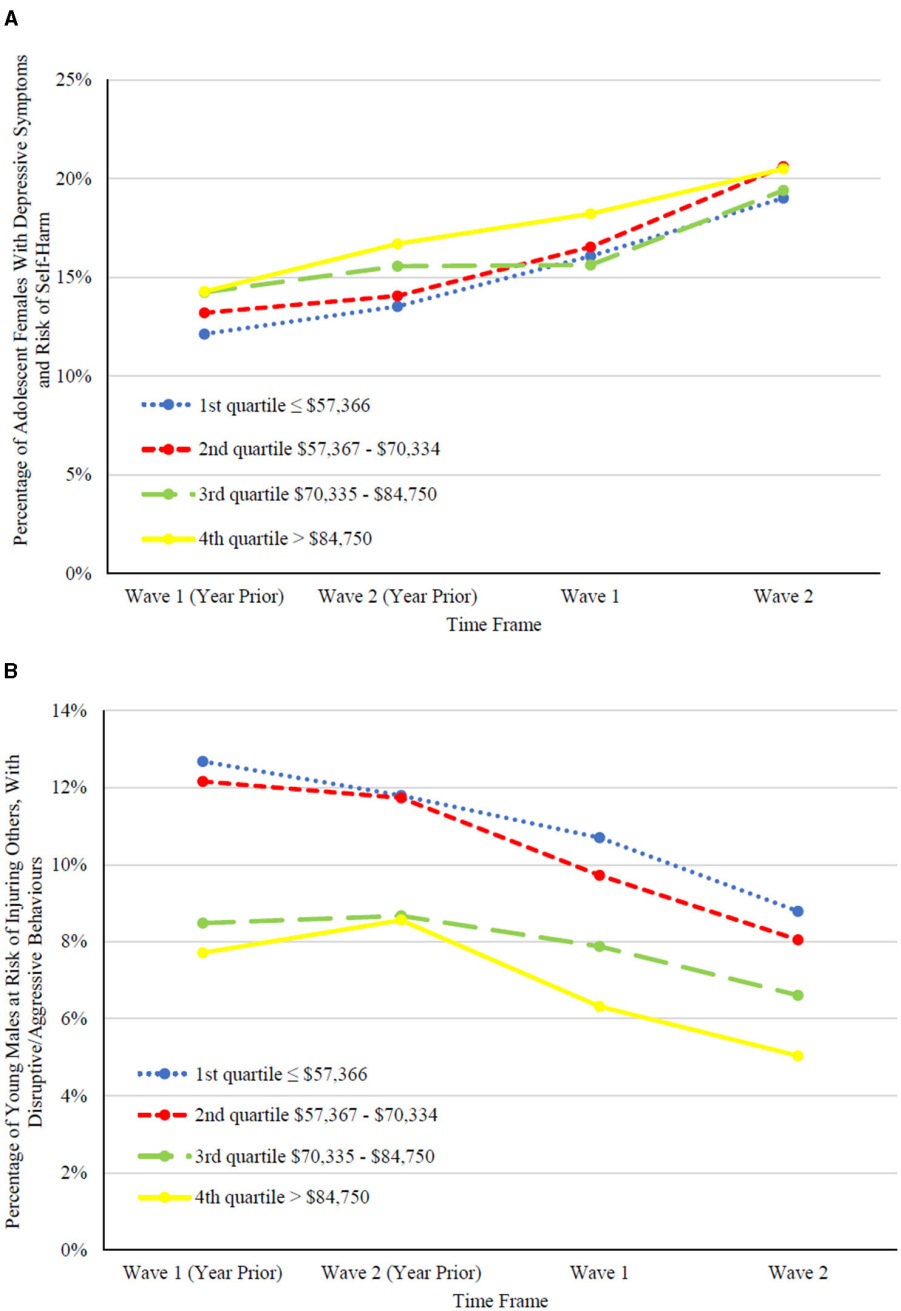
Examples of significant symptom changes observed in children and youth by income quartile. **(A)** represents the percentage of assessments for adolescent females, 12 years or older, with depressive symptoms, DSI ≥ 4, and risk of self-harm, RiSsK ≥ 1, by income quartile. **(B)** represents the percentage of assessments for young males, 11 years or younger, at risk for injuring others, RIO ≥ 3, with disruptive/aggressive behaviors, DABS ≥ 4, by income quartile.

Figures 2A,B show four periods of time (year prior to Wave 1, year prior to Wave 2, Wave 1, Wave 2) stratified by neighborhood income quartile for subgroups selected using some of the largest time-related differences. The first group are female clients aged 12 and older, with depressive symptoms (DSI 4 or greater) and risk of self-harm (RiSsK 1 or greater). The second group are male clients aged 11 and younger at risk of injuring others (RIO 3 or greater) and with disruptive/aggressive behavior (DABS 4 or greater). The first group are more prevalent in the higher half of neighborhood incomes (*p* = 0.0001), and the second group are more prevalent in the lower half of neighborhood incomes (*p* <0.0001). Over time, the first group shows significantly greater prevalence (*p* <0.0001) and the second group significantly lower prevalence (*p* <0.0001).

## Discussion

This study highlights an overall decline in the number of child and youth mental health assessments across 53 select mental health agencies in Ontario during the first wave of the pandemic, with some recovery during the second wave. Regarding the mental health presentations of children and youth during the COVID-19 pandemic, there were declines noted in the proportion of children exhibiting disruptive/aggressive behaviors and risk of harm to others, with increases in depressive symptoms and recent incidents of self-harm noted in both waves when compared to the year prior, with these findings remaining significant during the second wave when adjusted for factors such as age and sex. Moreover, it was in the second wave, when students had the option of attending in-person classes, that a higher number of significant differences were observed between the two waves, with notable increases in depressive symptoms and recent self-injurious attempts, alongside decreases in risk of injury to others and disruptive/aggressive behaviors from the first to second wave. We also observed significant increases in the proportion of children and youth experiencing domestic violence, along with parents having had a recent major life stressor, in both waves. Surprisingly, these changes were accompanied by declines in child protection service involvement in both waves.

These findings largely align with other previously cited international investigations noting concerning declines in children and youth accessing mental health services during periods of lockdown (29–38). When examining potential changes in mental health presentations within hospitals and emergency department settings outside of Canada, Ougrin et al. (36) noted an increase in proportion of children and youth presenting with self-harm while four other studies (31–33, 37) noted no significant difference or a decline in the proportion of children and youth in their sample presenting with self-harm and suicidality during the pandemic when compared to comparable pre-pandemic time frames. However, at the time of our investigation, we were unable to locate any studies examining specific changes in mental health presentations compared to overall assessment volumes to community mental health settings or emergency rooms within Canada. Due to a myriad of factors such as the scarcity of the research in this area, differences in referral pathways and nature of services within different settings providing mental health services to children and youth (e.g., community mental health, emergency, psychiatric hospitals), differences in government regulations across settings and areas served, differences in the selection of a comparison frame, and service equity and differential client access to services, any similarities and differences noted here should be interpreted with caution and no firm conclusions about specific changes in treatment-seeking children's mental health presentations during the pandemic can be drawn at this current time.

However, the sharp decline in children and youth accessing services across multiple studies and settings warrants attention. Considering the critical role that schools and educational childcare settings play in the identification of the mental health needs of children and service referrals to mental health agencies in Ontario (9, 12), it is possible that a large proportion of these findings are related to school and childcare setting closures. Educators are in a unique position to identify mental health needs of specific students (75, 76) and are often one of the first professionals that parents contact with respect to concerns about their children's mental health needs (77). Moreover, teachers tend to report higher rates of problematic symptoms than parents alone (75). Schools are also often one of the top referral sites to child protection services for young children in Ontario (78). In the absence of in-person classes, it is possible that the various needs of students are not being identified through online modules to the same level of efficiency as before. Moreover, aside from schools, primary care settings in Canada also play a critical role in identifying and addressing the mental health needs of children, including providing referrals to mental health specialists (79). Parents might have been hesitant to access primary care settings during periods of lockdown due to fears of contracting the virus or public messaging discouraging in-person service access, aside from cases of emergencies (35). In relation to online service delivery, concerns of confidentiality of sessions, privacy of client information, and clinician competency and training in online service provision also have the potential to impact the uptake of these services during this time (80–83).

Interestingly, assessment volumes in low-income neighborhoods declined more than those in high income neighborhoods during the second wave of the pandemic. Considering that families in these neighborhoods are at the greatest risk for exposure to pandemic-related stressors (42, 43), this decline likely does not reflect a decline in need for such services within these communities. While many mental health agencies shifted to online service delivery during the pandemic (80), this mode of service delivery may not be accessible to all families, especially those living in low-income neighborhoods who might not have access to the tools and technology required for online assessments (42, 84). For example, children living within families with lower incomes are disproportionately less likely to have access to internet at home, and more than one device per household, for the use of online services (80, 84). Moreover, considering that areas with the highest levels of material deprivation (e.g., educational attainment, income, and housing) in Ontario experienced disproportionately higher rates of transmission (85), it is also possible that families within lower income neighborhoods were more hesitant to send their children back to school during the second wave (86, 87). However, due to the lack of empirical data exploring the intersectionality of income and access to child and youth mental health services during the pandemic in Ontario, these hypotheses should be interpreted with caution.

In the second wave, disruptive/aggressive behaviors, risk of harm to others, and youth justice involvement also declined when compared to both one year prior and the first wave. These behaviors have been associated with peer rejection and victimization in previous studies (88–90). School closures might present with fewer opportunities for peer socialization and victimization, thus resulting in a reduction in the presentation of these behaviors. Given that teachers are in a unique position to identify such behavioral issues that might emerge within the classroom (76, 91), it is possible that some of these behaviors are currently under-detected and will re-emerge once all children have resumed in-person learning.

In our sample of treatment-seeking children and youth, we also noted a significant increase in depressive symptoms and recent self-injurious behaviors in the second wave when compared to the year prior and the previous wave. Self-harm behaviors can be used as coping strategies which alleviate feelings of negative affect, such as anger, depression, loneliness, and frustration (92, 93). These emotions may be experienced at heightened levels throughout the COVID-19 pandemic (10, 11, 27), becoming further exacerbated as the pandemic continues to unfold. Moreover, the length of social isolation may be more detrimental to youth psychological well-being, as measured by anxiety and depression, than the severity of the isolation itself (27).

There is also some evidence to suggest resilience in certain families during the pandemic. In our sample, there was an overall decline in the proportion of families reporting caregiver distress in both waves and the family reporting feeling overwhelmed in the second wave. Canada was able to implement its financial relief program, the Canadian Emergency Relief Benefit, early in the pandemic (94), which may have mitigated some of the potential negative impacts of unemployment and financial stress on caregiver stress and coping in treatment-seeking families. Moreover, some children might be benefiting from increased quality time and attention from caregivers (26, 95). For children who experience school as a major source of distress (e.g., bullying), school closures may also come as a relief for both the parent and child and may allow for these children to spend more time in a safer and more relaxed environment (26, 28).

### Limitations, Implications, and Future Directions

The data in this study only consists of treatment-seeking children from select mental health agencies within the Province of Ontario, and hence these findings might not be representative of all children in the province, those seeking secondary mental health services outside of these agencies or within emergency settings, or those in other regions that may be differently impacted by the pandemic and related government restrictions and closures. We also want to acknowledge that this investigation utilized an indirect measure of family income to analyze income related trends in child and youth mental health referrals and needs. Therefore, the subset of our findings that account for the impact of socioeconomic status on these mental health trends reflect aggregated neighborhood-level trends, not individual-level data.

Moreover, in this study, we classified pandemic period data from March 2020 to January 2021 into two distinct waves, discounting summer months. This resulted in shorter pandemic comparison periods than our previous eight-month investigation (13). The difference in timeframe, and subsequently the number of participants, resulted in this paper not capturing the modest increase in anxiety reported in our previous study (13). It is important to note that due to school closures, it is likely that our investigations are also not representative of the full scope of changes in domestic violence cases in the province. Furthermore, the data represents a limited time period as it only considers the first two waves of the pandemic in Ontario. Future research is needed to examine both the referral trends and the various mental health presentations of children not only throughout all potential waves of the pandemic in Ontario, but also once the pandemic subsides (10, 96).

We also want to address that in this study, we did not directly measure the COVID-19 impact on children, families, and agencies. While we provide a summary of children's mental health referral trends and presentations, as well as socioeconomic and familial factors related to children's mental health (e.g., caregiver distress or financial trade-offs), during the first two waves of the pandemic, future studies are needed to examine the ways that these socioeconomic and familial factors, and any potential pandemic-related changes in these factors, might interact with children's mental health presentations during periods of lockdown. Moreover, the direct impacts of COVID-19 pandemic on individual participants and their families (e.g., exposure to the illness, caregiver unemployment, or level of social isolation) that might impact children and adolescent' mental health needs during this time are not measured in our study and warrant future investigation. We also did not examine the ways that lockdowns and online service provision might impact service availability. Therefore, empirical research examining factors related to mental health service availability that might impact referrals to mental health services in the context of lockdown, where in-person services might not be available for long periods of time, is required to better understand the needs of this population during pandemics.

Despite these limitations, this study utilized a large sample of thousands of children and youth from the Province of Ontario, highlighting the imminent need for research into factors that impact service utilization of children and youth during this time. While these analyses focus on the proportions of individuals with the measured characteristics of interest, it is also important to pause and consider the absolute decline in the volumes of individuals with serious mental health characteristics who are observed seeking service during the pandemic. It is highly unlikely that the pandemic resulted in fewer persons with these needs, but rather this absolute decline was due to a combination of factors, including fewer cases identified and referred by schools and primary care, hesitancy of clients or families in seeking care due to risk of COVID exposure, and the child/youth mental agencies altered ability to offer services in the same way during the pandemic. One can infer that the true number of needy cases is at least as high as before the pandemic (if not higher), which suggests that a large number of cases are going without contact with the formal system. Our finding that cases newly referred to agencies declined more than existing cases hints at the large number of families with new onset conditions that would have been accessing services, if not for the pandemic. There might be an exponential increase in services needs and utilization of children and youth once public spaces and schools completely re-open in person and mental health services resume as normal (12).

Overall, this reduction in mental health utilization also reflects the need for the prioritization of the implementation of evidence-based assessment tools that can be easily transferable to online services (71, 73). Further, increased structural and financial support for children, youth, and their families within vulnerable communities during this time is needed to ensure appropriate, and timely access to services and devices required for online service delivery (22). Lastly, professional development opportunities and support for teachers to help increase their competency in identifying and supporting children presenting with mental health needs, or at risk for harm (24), during periods of lockdown is of upmost importance.

## Conclusion

The present study provides insight into the mental health needs and referral trends to mental health services of a subset of treatment-seeking children across select mental health agencies within province of Ontario during the first two waves COVID-19 pandemic. Overall, our investigation found a decline in assessments of children at mental health agencies in Ontario compared to pre-pandemic periods, with the greatest decline in assessments seen in low-income neighborhoods during the first wave. This was coupled with an increase in the proportion of certain mental health concerns, such as depression and self-harm, and a decrease in the proportion of others, such as disruptive and aggressive behavior and risk of injury to others. Overall, the shifts in these client characteristics during the pandemic needs to be considered along with the overall decline in treatment-seeking cases, which suggests large numbers of untreated cases during the pandemic. Moreover, these findings provide insight for clinicians and researchers into the evolving needs of treatment-seeking children and youth during periods of uncertainty and duress.

## Data Availability Statement

The data analyzed for this study was de-identified standard client care data that was obtained from the interRAI database. This data is made available to interRAI Research Fellows and their students, for research purposes under an existing license agreement with the Canadian Institute for Health Information. Under this agreement, this data may not be transmitted to third parties; hence, it cannot be included as Supplementary Materials for this study. Those who would like to access the data obtained for this study can apply to the Canadian Institute for Health Information for access to this data. Requests to access these datasets should be directed to https://www.cihi.ca/en/access-data-and-reports/make-a-data-request.

## Ethics Statement

Ethics clearance for secondary analyses of interRAI data gathered by other organizations was obtained from Western University (REB #106415).

## Author Contributions

All authors contributed to the formulation of ideas presented in this study and provided critical feedback to the manuscript. SS drafted the first version of the materials, procedure, and methodology sections. AV and JV drafted the first version of the introduction and discussion sections collaboratively. JP conducted all the analyses. All authors contributed to the article and approved the submitted version.

## Funding

This study was partially funded by the Public Health of Canada Grant 1617-HQ-000050.

## Conflict of Interest

The authors declare that the research was conducted in the absence of any commercial or financial relationships that could be construed as a potential conflict of interest. The handling editor JF declared a past coauthorship/collaboration with one of the authors SS.

## Publisher's Note

All claims expressed in this article are solely those of the authors and do not necessarily represent those of their affiliated organizations, or those of the publisher, the editors and the reviewers. Any product that may be evaluated in this article, or claim that may be made by its manufacturer, is not guaranteed or endorsed by the publisher.

## Footnotes

^1^Stewart SL, Celebre A, Hirdes JP, Poss JW. Risk of injury to others: the development of an algorithm to identify youth at high-risk of violence perpetration within the mental health system. (under review).

## Supplementary Material

Supplementary Figure 1 This represents 14-day averages of COVID-19 cases in Ontario (92) and our selected COVID-19 wave boundaries.

Supplementary Table 1 This includes unadjusted values for the outcome scales and select measures utilized in this study, during the first two waves of the pandemic, when compared to one year prior and each other.
