# Supplementary figures and images for: Following the Epidemic Waves: Child and Youth Mental Health Assessments in Ontario Through Multiple Pandemic Waves
Source: Front Psychiatry. 2021 Nov 17;12:730915. doi: 10.3389/fpsyt.2021.730915 (PMC8635704; doi:10.3389/fpsyt.2021.730915)

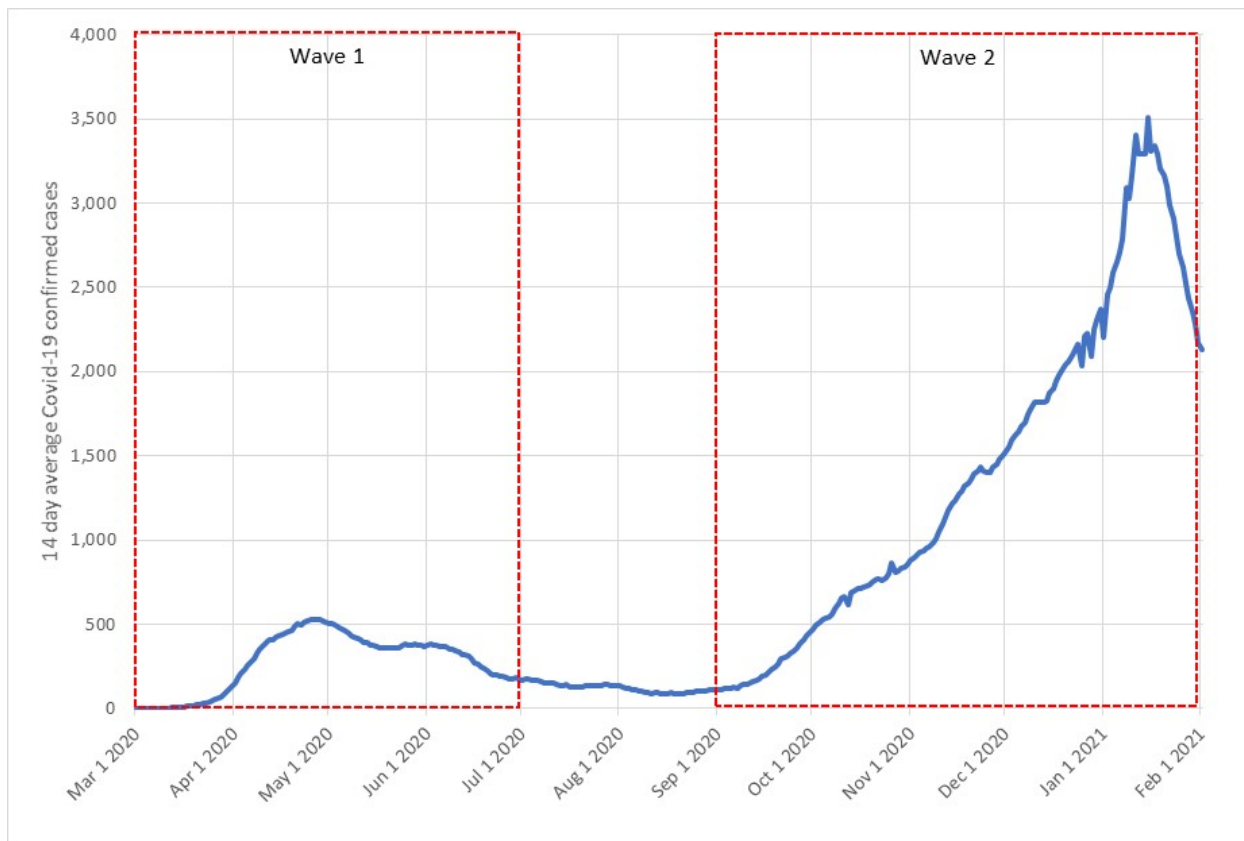

Supplement: Supplementary Figure 1 — This represents 14-day averages of COVID-19 cases in Ontario (92) and our selected COVID-19 wave boundaries. [file Data_Sheet_1.PDF]
