# Supplementary material for: Following the Epidemic Waves: Child and Youth Mental Health Assessments in Ontario Through Multiple Pandemic Waves
Source: Front Psychiatry. 2021 Nov 17;12:730915. doi: 10.3389/fpsyt.2021.730915 (PMC8635704; doi:10.3389/fpsyt.2021.730915)
Supplement: Supplementary Table 1 — This includes unadjusted values for the outcome scales and select measures utilized in this study, during the first two waves of the pandemic, when compared to one year prior and each other. [file Table_1.docx]

**Supplementary Table 1**

*Outcome Scales and Selected Measures During Wave 1 and 2 of the COVID-19 Pandemic Compared to the Period 1 Year Prior - unadjusted*

| **Outcome Scales and Selected Measures** | **Wave 1 ^a^** | | | **Wave 2 ^b^** | | |
| --- | --- | --- | --- | --- | --- | --- |
|  | Period prior | COVID-19 period | *p* | Period prior | COVID-19 period | *p* |
| Risk of Suicide and Self Harm in Kids (RISSK) 1+ | 48.6% | **51.9%** | <.0001* | 49.2% | **52.1%** | <.0001* |
| Risk for Injury to Others (RIO) 1+ | **47.9%** | 43.8% | <.0001* | **48.1%** | 40.5% | <.0001* |
| Destructive/Aggressive Scale (DABS) 4+ | **39.3%** | 36.0% | <.0001* | **39.2%** | 33.5% | <.0001* |
| Depressive Symptom Inventory (DSI) 4+ | 47.4% | **49.3%** | 0.018* | 49.0% | **51.4%** | 0.001* |
| Anxiety Scale 3+ | 45.7% | 44.0% | 0.066 | **45.0%** | 43.1% | 0.017* |
| Hyperactivity/Distractibility Scale (HDS) 9+ | 30.2% | 29.1% | 0.129 | **30.9%** | 27.8% | <.0001* |
| Witnessed domestic violence within last month | 1.0% | 1.0% | 0.974 | 1.2% | 1.0% | 0.187 |
| Witnessed domestic violence within last year | 5.0% | 5.5% | 0.149 | 5.4% | 5.5% | 0.626 |
| Experienced sexual assault/abuse within last year | 2.2% | 2.3% | 0.808 | 2.3% | 2.3% | 0.895 |
| Experienced physical assault/abuse within last year | 4.3% | 4.6% | 0.430 | **4.6%** | 4.0% | 0.031* |
| Experienced emotional abuse within last year | 9.2% | 10.0% | 0.070 | 10.4% | 9.9% | 0.279 |
| Self-injurious attempt in the last month | 8.3% | 8.6% | 0.541 | 7.9% | **9.9%** | <.0001* |
| Referral as a result of involvement with youth justice system | 5.1% | 5.9% | 0.224 | **5.6%** | 3.8% | 0.001* |
| Street drug use (illegal or legal) last 14 days | 5.8% | 6.1% | 0.613 | 6.7% | 6.0% | 0.110 |
| Legal guardian mother or father only | 29.5% | 28.8% | 0.317 | **29.1%** | 27.3% | 0.007* |
| Current custody dispute | **5.3%** | 4.1% | 0.038* | 4.2% | 4.5% | 0.631 |
| Parent/primary caregiver expresses feelings of distress, anger, or depression | **30.6%** | 26.7% | 0.012* | **33.3%** | 25.2% | <.0001* |
| Family members report feeling overwhelmed | **35.0%** | 33.3% | <.0001* | **37.0%** | 31.6% | <.0001* |
| Parent experienced major life stressor last 90 days | 23.5% | **27.6%** | 0.004* | 23.7% | 24.9% | 0.490 |
| Parental addiction in the last month | 3.9% | 3.5% | 0.214 | 3.7% | 3.6% | 0.715 |
| Limited funds resulted in child/youth or parent making economic trade-offs | 3.4% | 2.6% | 0.119 | **3.6%** | 2.5% | 0.011* |
| Child protection services received last 90 days | **21.1%** | 17.1% | 0.000* | **19.3%** | 16.5% | 0.003* |

*Note.* Bolded numbers denote the period with the higher % to indicate the direction of the change from the COVID-19 period and the period prior, where the difference is statistically significant.

^a^ Wave 1 represents March 2020 to June 2020 (4 months).

^b^ Wave 2 represents September 2020 to January 2021 (5 months).

* *p* < .05.
